# A Novel Case of Multisystem Inflammatory Syndrome in Children Associated With Influenza

**DOI:** 10.1002/ccr3.70871

**Published:** 2025-09-10

**Authors:** Nazanin Zibanejad, Niloofar Mohkamkar

**Affiliations:** ^1^ Department of Pediatric Intensive Care, Imam Hossein Children Hospital Isfahan University of Medical Sciences Isfahan Iran; ^2^ Child Growth and Development Research Center, Research Institute for Primordial Prevention of Non‐Communicable Disease Isfahan University of Medical Sciences Isfahan Iran; ^3^ Department of Pediatrics, School of Medicine Isfahan University of Medical Sciences Isfahan Iran

**Keywords:** case report, children, COVID‐19, influenza, multisystem inflammatory syndrome

## Abstract

It is critical to consider MIS‐C diagnosis following viral infections in the pediatric population, especially when symptoms cannot be fully explained by other conditions.

## 1 Introduction

Multisystem Inflammatory Syndrome in Children (MIS‐C) was first described in the United Kingdom in April 2020, when an alarm was raised by the Pediatric Intensive Care Society regarding a significant rise in critically ill children exhibiting hyperinflammatory shock and signs of SARS‐CoV‐2 infection. The condition was initially termed Pediatric Multisystem Inflammatory Syndrome (PIMS‐TS) by the Royal College of Pediatricians and Child Health (RCPCH) [1]. Early reports demonstrated clinical features resembling those of Kawasaki disease (KD), toxic shock syndrome (TSS), and macrophage activation syndrome (MAS), alongside secondary hemophagocytic lymphohistiocytosis (HLH) [2, 3, 4]. In response, both the Centers for Disease Control and Prevention (CDC) [5] and the World Health Organization (WHO) [6] issued diagnostic criteria for MIS‐C.

The emergence of MIS‐C raised concerns regarding its differentiation from other viral and bacterial infections, particularly during the first winter of the COVID‐19 pandemic, when the overlap between SARS‐CoV‐2 and other respiratory viruses was not fully understood [7]. While these concerns were largely alleviated as it became evident that COVID‐19 was the predominant respiratory virus, confusion regarding the clinical presentation of MIS‐C persisted. The syndrome shares clinical features with a range of conditions, including KD, septic shock, bacterial infections, and various rheumatological disorders. Indeed, the diagnostic criteria for MIS‐C can overlap significantly with other hyperinflammatory conditions characterized by prolonged fever. This case highlights the challenges associated with distinguishing MIS‐C from other similarly presenting diseases, underscoring the complexity of its clinical diagnosis in our clinical practice.

## 2 Case History

We present a case of a 5.5‐year‐old female patient with no significant medical history, who was admitted to the pediatric intensive care unit (PICU) with a 2‐day history of fever, vomiting, and rhinorrhea. On the morning of admission, she developed increased lethargy and a persistent cough, prompting her transfer to the hospital by ambulance.

Upon arrival, the patient was lethargic, with miotic pupils that were sluggishly reactive to light. She responded to mild painful stimuli but communicated with unintelligible speech. Oxygen saturation was critically low at 80%, and she exhibited mild intercostal retractions and abundant pharyngeal secretions. Notably, there were no signs of hypoperfusion or shock.

## 3 Investigations and Treatment

The initial fever (*T* = 38°C) and altered mental status prompted the initiation of empirical antibiotic therapy with ceftriaxone and vancomycin. Given abnormal liver enzyme levels (AST = 308, ALT = 1100, ALP = 998, PT = 20.7, PTT = 31, INR = 1.37), ceftriaxone was substituted with cefotaxime.

A brain CT scan was performed immediately upon admission and again 24 h later due to the persistence of altered consciousness; both scans were reported as normal. Similarly, an electroencephalogram (EEG) showed no abnormal findings.

Despite aggressive management, the patient's respiratory distress worsened, and her oxygen saturation continued to decline, necessitating the initiation of noninvasive ventilation (NIV). Over the next 48 h, the patient's condition deteriorated further, with increased nasal flaring, severe respiratory distress, and progressive involvement of the lung parenchyma. Consequently, she required intubation and mechanical ventilation. Given the seasonal context and the prevailing viral outbreak, along with a viral pattern noted on the chest X‐ray, Tamiflu was administered from the outset. A viral panel obtained from a non‐bronchoscopic bronchoalveolar lavage (NBBAL) sample confirmed the presence of Influenza A by PCR. We sent one NBBAL for evaluation of bacterial infection, which was negative. Two BACKTEC×2 peripheral blood samples were also sent, which were also negative, ruling out bacterial infection.

The patient's laboratory results revealed markedly elevated inflammatory markers: ProBNP (3565 pg/mL), ESR (85 mm/h), CRP (29 mg/dL), and ferritin (1182 ng/mL), alongside the positive influenza PCR result (Table 1).

**TABLE 1:** Clinical features of the presented case.

Parameters	Baseline	After intervention
WBC; ×10^9^/L	5.9	9.3
Lymphocytes; %	47	38
Neutrophils; %	41	65
Hb; g/dL	9.7	12.4
PLT; ×10^9^/L	80	281
ALT; U/L	1100	27
AST; U/L	308	37
LDH; U/L	453	256
CPK; μg/L	5071	27
Alb; g/dL	1.8	4.3
PT; s	20.7	12.5
PTT; s	31	21
INR	1.37	1
Troponin; ng/mL	137	—
Procalcitonin; ng/mL	10.19	—
IL‐6; pg/mL	5	—
Ferritin; ng/mL	1182	732
Fibrinogen; mg/dL	386	134
CRP; mg/dL	29	< 3
ESR; mm/h	85	60
ProBNP; pg/mL	3565	

Respiratory pathogen screening PCR including Influenza A virus, Influenza B virus, Influenza A(H1N1) virus, Rhinovirus, Coronavirus NL63, Coronavirus 229E, Coronavirus OC43, Coronavirus HKU1, Parainfluenza 1, 2, 3, 4, Metapneumoviruses A/B, Bocavirus, Mycoplasma pneumonia, RSV A/B, Adenovirus, Enterovirus, and Parechovirus was checked, only Influenza A virus was positive.

## 4 Conclusion and Results

Given the ongoing decline in consciousness and the multisystem involvement (respiratory, hepatic, and cardiovascular), a diagnosis of MIS‐C was considered in the context of influenza infection. It should be noted that, according to previous studies, although severe influenza can justify an increase in inflammatory factors, a significant increase in ferritin (Ferritin: 1182 ng/mL) and CRP (CRP: 29) can also occur in the context of severe inflammatory diseases such as MIS‐C and HLH [8, 9].

Following consultations with rheumatology and infectious disease specialists, the patient received two pulses of 10 mg/kg methylprednisolone and 2 g/kg intravenous immunoglobulin (IVIG). Here, it can also be stated that the response to treatment with IVIG and pulsed corticosteroid is also evidence confirming the diagnosis of this disease.

As the patient developed acute respiratory distress syndrome (ARDS), with increasing ventilator settings due to concern for ventilator‐associated pneumonia (VAP), antibiotic therapy was broadened to include meropenem, vancomycin, and amikacin. Echocardiographic findings revealed mild tricuspid regurgitation (TR), mild mitral regurgitation (MR), mildly reduced left ventricular (LV) systolic function, and minimal pericardial effusion (PE).

Over the subsequent days, the patient's consciousness level gradually improved. The ventilator settings were gradually reduced, and her fever subsided. Approximately 11 days after intubation, she was successfully extubated and transitioned to NIV. As sedation was progressively tapered, the patient regained full consciousness, communicated appropriately, and exhibited increasing alertness.

In the days that followed, the patient's inflammatory markers decreased, and she was weaned off supplemental oxygen. By the time of discharge, her clinical status had markedly improved, with full recovery of consciousness and resolution of fever. Follow‐up echocardiography showed minimal MR and TR, improved LV systolic function, normal coronary arteries, and an ejection fraction (EF) of 65%. The patient was discharged from the PICU in stable condition.

## 5 Discussion

Multisystem Inflammatory Syndrome in Children was first recognized in the United Kingdom as a hyperinflammatory shock syndrome associated with COVID‐19, presenting similarly to KD in a cohort of eight children [10]. As similar cases were reported globally, diagnostic criteria were subsequently established by the CDC and the WHO [5, 6]. These criteria encompass persistent fever (T ≥ 38; during ≥ 24 h or documentary fever in hospital), elevated inflammatory markers (CRP, Ferritin, ESR. LDH, Interleukin‐6), involvement of at least two organ systems, hospitalization due to severe disease, exclusion of other diagnoses, and evidence of SARS‐CoV‐2 infection [11]. MIS‐C, characterized by fever, elevated inflammatory markers, and multisystem organ involvement, has predominantly been observed in children following SARS‐CoV‐2 infection. However, it remains an exceptional diagnosis, requiring careful differential diagnosis and thorough investigation.

It is worth noting that, given that the patient's condition met the above criteria, the T‐cell test was not performed for this patient; however, this test is rarely found in Iran and we did not have access to it.

Dworsky et al. [12] first highlighted the association between prolonged fever, gastrointestinal symptoms, and MIS‐C, noting that many of the patients initially diagnosed with MIS‐C were later found to have bacterial enteritis. Similarly, Kara et al. [1] reported two cases that were initially diagnosed as MIS‐C, only for subsequent blood cultures to reveal the presence of gram‐negative bacteria. These cases underscore the importance of maintaining a broad differential diagnosis, particularly given the overlap of MIS‐C with other infectious and inflammatory conditions.

The treatment of MIS‐C poses significant challenges, especially as it shares clinical features with other common diseases. In cases presenting with fever, multisystem organ involvement, and elevated inflammatory markers, empiric antibiotic therapy should be initiated to cover potential bacterial pathogens. If there is a high clinical suspicion of MIS‐C, the antibiotic regimen may need to be extended.

However, with close monitoring and review of culture results, antibiotics can be discontinued promptly if a bacterial infection is ruled out.

Viral infections, including influenza, have been shown to predispose children to secondary infections, such as streptococcal infections, which can complicate the clinical course. This potential for secondary bacterial infections is of particular concern in the context of the global influenza pandemic, where influenza‐induced immune dysregulation may facilitate the development of bacterial superinfections. The increased frequency of such secondary infections during the influenza season further complicates the diagnosis and management of MIS‐C.

In our patient, the presentation of 2 days of persistent fever, elevated inflammatory markers (CRP, ferritin, fibrinogen, LDH, and procalcitonin), respiratory involvement, altered consciousness, high liver enzymes, gastrointestinal symptoms, and lymphopenia was highly suggestive of MIS‐C. The delay in initiating steroid therapy was primarily due to the combination of high fever and marked inflammatory markers, which ultimately supported the diagnosis of MIS‐C. Despite these findings, the patient's platelet count was below the normal range, a feature that is often observed in MIS‐C, alongside the noted lymphopenia.

The detection of Influenza A in the respiratory virus panel was not unexpected in the context of seasonal influenza, yet it presented a diagnostic challenge given the clinical overlap with MIS‐C. Influenza A typically causes acute fever, headache, muscle pain, fatigue, and respiratory symptoms, such as cough, sore throat, and rhinitis. In younger children, gastrointestinal manifestations including diarrhea, nausea, vomiting, and anorexia are also common, all of which can overlap with the clinical presentation of MIS‐C. While influenza can cause prolonged fever, it rarely induces a rash, and reports of co‐occurrence with KD are limited, with only a few cases documenting coinfection [2, 3, 4, 13]. The appearance of a red tongue, a hallmark of MIS‐C, has not been documented in influenza infections, making its occurrence in our case all the more distinctive.

Lymphopenia is one of the most consistent laboratory findings in MIS‐C. A multicenter study involving 614 MIS‐C patients found that over half of the cases had CRP levels exceeding 100 mg/L, highlighting the substantial elevation of inflammatory markers in this syndrome [7], a finding that aligns with our patient's laboratory results.

A recent report described a 10‐year‐old girl with fever, headache, vomiting, and weakness, where procalcitonin levels were mildly elevated (3.7 μg/L), and CRP was moderately increased (41 mg/L). Despite these relatively modest increases, the patient met the CDC case criteria for MIS‐C, prompting the initiation of MIS‐C‐specific therapy alongside empirical antibiotics. The patient showed rapid clinical improvement following fluid resuscitation and antibiotic therapy. Influenza A was detected on the respiratory viral panel, and a subsequent *Streptococcus pyogenes* infection was confirmed by throat culture, leading to the final diagnosis of a bacterial infection. As a result, MIS‐C treatment was discontinued, and targeted antibiotic therapy continued [12].

In our case, the detection of Influenza A in the respiratory viral panel, in conjunction with the clinical and laboratory features that met the criteria for MIS‐C, prevented the exclusion of MIS‐C. Consequently, treatment with IVIG and steroids was initiated, resulting in complete resolution of symptoms in the following days.

Given the clinical significance of MIS‐C, particularly in the pediatric population, it is critical to consider this diagnosis following viral infections, including influenza, especially when symptoms cannot be fully explained by other conditions. The need for timely recognition and appropriate treatment is paramount, as early intervention can lead to significant improvements in patient outcomes.

As awareness of MIS‐C grows, it is likely that more cases will be identified, not only following COVID‐19 but also in the context of other viral infections. Therefore, clinicians must remain vigilant for its occurrence and be prepared to initiate appropriate treatment promptly.

In our case, this hyperinflammation was due to severe influenza virus disease, which led to multisystem involvement, and ultimately the diagnosis of MIS‐C was proposed for her.

## Author Contributions

**Nazanin Zibanejad:** resources, supervision, validation, writing – review and editing. **Niloofar Mohkamkar:** conceptualization, data curation, investigation, writing – original draft.

## Consent

Written informed consent from the patient was obtained according to journal guidelines.

## Conflicts of Interest

The authors declare no conflicts of interest.

## Data Availability

Data are made available with the coordination of the corresponding author.
